# Counterexample-Guided Prophecy for Model Checking Modulo the Theory of Arrays

**DOI:** 10.1007/978-3-030-72016-2_7

**Published:** 2021-03-01

**Authors:** Makai Mann, Ahmed Irfan, Alberto Griggio, Oded Padon, Clark Barrett

**Affiliations:** 8 Eindhoven University of Technology, Eindhoven, The Netherlands; 9 Aalborg University, Aalborg East, Denmark; 10 Stanford University, Stanford, USA; 11 Fondazione Bruno Kessler, Trento, Italy; 12 VMware Research, Palo Alto, USA

## Abstract

We develop a framework for model checking infinite-state systems by automatically augmenting them with auxiliary variables, enabling quantifier-free induction proofs for systems that would otherwise require quantified invariants. We combine this mechanism with a counterexample-guided abstraction refinement scheme for the theory of arrays. Our framework can thus, in many cases, reduce inductive reasoning with quantifiers and arrays to quantifier-free and array-free reasoning. We evaluate the approach on a wide set of benchmarks from the literature. The results show that our implementation often outperforms state-of-the-art tools, demonstrating its practical potential.

## 1 Introduction

Model checking is a widely-used and highly-effective technique for automated property checking. While model checking finite-state systems is a well-established technique for hardware and software systems, model checking infinite-state systems is more challenging. One challenge, for example, is that proving properties by induction over infinite-state systems often requires the use of universally quantified invariants. While some automated reasoning tools can reason about quantified formulas, such reasoning is typically not very robust. Furthermore, just discovering these quantified invariants remains very challenging.

Previous work (e.g., [52]) has shown that prophecy variables can sometimes play the same role as universally quantified variables, making it possible to transform a system that would require quantified reasoning into one that does not. However, to the best of our knowledge, there has been no automatic method for applying such transformations. In this paper, we introduce a technique we call *counterexample-guided prophecy.* During the refinement step of an abstraction-refinement loop, our technique automatically introduces prophecy variables, which both help with the refinement step and may also reduce the need for quantified reasoning. We demonstrate the technique in the context of model checking for infinite-state systems with arrays, a domain which is known for requiring quantified reasoning. We show how a standard abstraction for arrays can be augmented with counterexample-guided prophecy to obtain an algorithm that reduces the model checking problem to quantifier-free, array-free reasoning.

The paper makes the following contributions: i) we introduce an algorithm called **Prophecize** which uses history and prophecy variables to target a specific term at a specific time step of an execution, producing a new transition system that can effectively reason universally about that term; ii) we develop an automatic abstraction-refinement procedure for arrays, which leverages the **Prophecize** algorithm during the refinement step, and show that it is sound and produces no false positives; iii) we develop a prototype implementation of our technique; and iv) we evaluate our technique on four sets of model checking benchmarks containing arrays and show that our implementation outperforms state-of-the-art tools on a majority of the benchmark sets.

## 2 Background

We assume the standard many-sorted first-order logical setting with the usual notions of signature, term, formula, and interpretation. A *theory* is a pair $\mathcal{T} = (\Sigma, \mathbf{I})$ where $\Sigma$ is a signature and $\mathbf{I}$ is a class of $\Sigma$-interpretations, the *models* of $T$. A $\Sigma$-formula $\varphi$ is *satisfiable* (resp., *unsatisfiable*) *in* $\mathcal{T}$ if it is satisfied by some (resp., no) interpretation in $\mathbf{I}$. Given an interpretation $\mathcal{M}$, a variable assignment $s$ over a set of variables $X$ is a mapping that assigns each variable $x \in X$ of sort $\sigma$ to an element of $\sigma^{\mathcal{M}}$, denoted $x^s$. We write $\mathcal{M}[s]$ for the interpretation that is equivalent to $\mathcal{M}$ except that each variable $x \in X$ is mapped to $x^s$. Let $x$ be a variable, $t$ a term, and $\phi$ a formula. We denote with $\phi\{x \mapsto t\}$ the formula obtained by replacing every free occurrence of $x$ in $\phi$ with $t$. We extend this notation to sets of variables and terms in the usual way. If $f$ and $g$ are two functions, we write $f \circ g$ to mean functional composition, i.e., $f \circ g(x) = f(g(x))$.

Let $\mathcal{T}_A$ be the standard theory of arrays [50] with extensionality, extended with constant arrays. Concretely, we assume sorts for arrays, indices, and elements, and function symbols *read*, *write*, and *constarr*. Here and below, we use $a$ and $b$ to refer to arrays, $i$ and $j$ to refer to array indices, and $e$ and $c$ to refer to array elements, where $c$ is also restricted to be an interpreted constant. The theory contains the class of all interpretations satisfying the following axioms:

$$\begin{aligned} \forall\, a, i, j, e.\; i = j &\implies \mathit{read}(\mathit{write}(a, j, e), i) = e \;\wedge \\ i \neq j &\implies \mathit{read}(\mathit{write}(a, j, e), i) = \mathit{read}(a, i) \end{aligned} \tag{write}$$

$$\forall\, a, b.\; (\forall\, i.\; \mathit{read}(a, i) = \mathit{read}(b, i)) \implies a = b \tag{ext}$$

$$\forall\, i.\; \mathit{read}(\mathit{constarr}(c), i) = c \tag{const}$$

**Symbolic Transition Systems and Model Checking.** For generality, assume a background theory $\mathcal{T}$ with signature $\Sigma$. We will assume that all terms and formulas are $\Sigma$-terms and $\Sigma$-formulas, that entailment is entailment modulo $\mathcal{T}$, and interpretations are $\mathcal{T}$-interpretations. A symbolic transition system (STS) $\mathcal{S}$ is a tuple $\mathcal{S} := \langle X, I, T \rangle$, where $X$ is a finite set of state variables, $I(X)$ is a formula denoting the initial states of the system, and $T(X, X')$ is a formula expressing a transition relation. Here, $X'$ is the set obtained by replacing each variable $x \in X$ with a new variable $x'$ of the same sort. Let $\mathit{prime}(x) = x'$ be the bijection corresponding to this replacement. We say that a variable $x$ is *frozen* if $T \models x' = x$. When the state variables are obvious, we will often drop $X$.

A state $s$ of $\mathcal{S}$ is a variable assignment over $X$. An *execution* of $\mathcal{S}$ of length $k$ is a pair $\langle \mathcal{M}, \pi \rangle$, where $\mathcal{M}$ is an interpretation and $\pi := s_0, s_1, \ldots, s_{k-1}$ is a *path* of length $k$, a sequence of states such that $\mathcal{M}[s_0] \models I(X)$ and $\mathcal{M}[s_i][s_{i+1} \circ prime^{-1}] \models T(X, X')$ for all $0 \leq i < k-1$. When reasoning about paths, it is often convenient to have multiple copies of the state variables $X$. We use $X@n$ to denote the set of variables obtained by replacing each variable $x \in X$ with a new variable called $x@n$ of the same sort. We refer to these as *timed* variables. A state $s$ is *reachable* in $\mathcal{S}$ if it appears in a path of some execution of $\mathcal{S}$. We say that a formula $P(X)$ is an *invariant* of $\mathcal{S}$, denoted by $\mathcal{S} \models P(X)$, if $P(X)$ is satisfied in every reachable state of $\mathcal{S}$ (i.e., for every execution $\langle \mathcal{M}, \pi \rangle$, $\mathcal{M}[s] \models P(X)$ for each $s$ in $\pi$). The *invariant checking problem* is, given $\mathcal{S}$ and $P(X)$, to determine if $\mathcal{S} \models P(X)$. A *counterexample* is an execution $\langle \mathcal{M}, \pi \rangle$ of $\mathcal{S}$ of length $k$ such that $\mathcal{M}[s_{k-1}] \not\models P(X)$. If $I(X) \models \phi(X)$ and $\phi(X) \wedge T(X, X') \models \phi(X')$, then $\phi(X)$ is an *inductive* invariant. Every inductive invariant is an invariant (by induction over path length). In this paper we focus on model checking problems where $I$, $T$ and $P$ are quantifier-free. However, a *quantified inductive invariant* might still be necessary to prove a property of the system.

*Bounded Model Checking* (BMC) is a bug-finding technique which attempts to find a counterexample for a property, $P(X)$, of length $k$ for some finite $k$ [9]. A single BMC query at bound $k$ for an invariant property uses a constraint solver to check the satisfiability of the following formula: $BMC(\mathcal{S}, P, k) := I(X@0) \wedge (\bigwedge_{i=0}^{k-1} T(X@i, X@(i+1))) \wedge \neg P(X@k)$. If the query is satisfiable, there is a bug.

**Counterexample-Guided Abstraction Refinement (CEGAR).** CEGAR is a general technique in which a difficult conjecture is tackled iteratively [44]. Algorithm 1 shows a simple CEGAR loop for checking an invariant $P$ for an STS $\mathcal{S}$. It is parameterized by three functions. The **Abstract** function produces an initial abstraction of the problem. It must satisfy the contract that if $\langle \widehat{S}, \widehat{P} \rangle =$ **Abstract**$(\mathcal{S}, P)$, then $\widehat{S} \models \widehat{P} \implies \mathcal{S} \models P$. The next function is the **Prove** function. This can be any (unbounded) model-checking algorithm that can return counterexamples. It checks whether a given property $P$ is an invariant of a given STS $\mathcal{S}$. If it is, it returns with *proven* set to true. Otherwise, it returns a bound $k$ at which a counterexample exists. The final function is **Refine**. It takes the abstracted STS and property together with a bound $k$ at which a known counterexample for the abstract STS exists. Its job is to refine the abstraction until there is no longer a counterexample of size $k$. If it succeeds, it returns the new STS and property. It fails if there is an actual counterexample of size $k$ for the concrete system. In this case, it sets the return value *refined* to false.

**Auxiliary variables.** We finish this section with relevant background on *auxiliary* variables, a crucial part of the refinement step described in Sec. 4. Auxiliary variables are new variables added to the system which do not influence its behavior (i.e., the reduct to the old set of variables of any reachable state in the new system is a reachable state in the old system), but may assist in proofs. There are two main categories of auxiliary variables we consider: *history* and

**Algorithm 1 STS-CEGAR**($\mathcal{S} \coloneqq \langle X, I, T\rangle$, $P$)

```
⟨⟨X̂, Î, T̂⟩, P̂⟩ ← Abstract(S, P)
while true do
    ⟨k, proven⟩ ← Prove(⟨X̂, Î, T̂⟩, P̂)                        // try to prove
    if proven then return true                               // property proved
    ⟨⟨X̂, Î, T̂⟩, P̂, refined⟩ ← Refine(⟨X̂, Î, T̂⟩, P̂, k)          // try to refine
    if ¬refined then return false                      // found counterexample
end while
```

*prophecy*. History variables, also known as *ghost state*, preserve a value, making its past value available in future states. Prophecy variables are the dual of history variables and provide a way to refer to a value that occurs in a future state. Abadi and Lamport formally characterized soundness conditions for the introduction of history and prophecy variables [1]. Here, we consider a simple, structured form of history variables.

**Definition 1.** *Let $\mathcal{S} = \langle X, I, T\rangle$ be an STS, $t$ a term whose free variables are in $X$, and $n > 0$, then **Delay**$(\mathcal{S}, t, n)$ returns a new STS and variable $\langle\langle X^h, I^h, T^h\rangle, h_t^n\rangle$, where $X^h = X \uplus \{h_t^1, \ldots, h_t^n\}$, $I^h = I$, and $T^h = T \cup \{h_t^{1\prime} = t\} \cup \bigcup_{i=2}^{n}\{h_t^{i\prime} = h_t^{i-1}\}$.*

The **Delay** operator makes the current value of a term $t$ available for the next $n$ states in a path. This is accomplished by adding $n$ new history variables and creating an assignment chain that passes the value to the next history variable at each state. Thus, $h_t^k$ contains the value that $t$ had $k$ states ago. The initial value of each history variable is unconstrained.

**Theorem 1.** *Let $\mathcal{S} = \langle X, I, T\rangle$ be an STS, $P$ a property, and **Delay**$(\mathcal{S}, v, n) = \langle \mathcal{S}^h, h_v^n\rangle$. Then $\mathcal{S} \models P$ iff $\mathcal{S}^h \models P$.*

We refer to [1] for a general proof which subsumes Theorem 1. In contrast to the general approach for history variables, we use a version of prophecy that only requires a single frozen variable. The motivation for this is that a frozen variable can be used in place of a universal quantifier, as the following theorem adapted from [52] shows.

**Theorem 2.** *Let $\mathcal{S} = \langle X, I, T\rangle$ be an STS, $x$ a variable in formula $P(X)$, and $v$ a fresh variable (i.e., not in $X$ or $X'$). Let $\mathcal{S}^p = \langle X \cup \{v\}, I, T \cup \{v' = v\}\rangle$. Then $\mathcal{S} \models \forall\, x.\, P(X)$ iff $\mathcal{S}^p \models P(X)\{x \mapsto v\}$.*

Theorem 2 shows that a universally quantified variable in an invariant can be replaced with a fresh symbol in a process similar to skolemization. The intuition is as follows. The frozen variable has the same value in all states, but it is uninitialized by $I$. Thus, for each path in $\mathcal{S}$, there is a corresponding path (i.e., identical except at $v$) in $\mathcal{S}^p$ for *every* possible value of $v$. This proliferation of paths plays the same role as the quantified variable in $P$. We mention here one more theorem from [52]. This one allows us to *introduce* a universal quantifier.

**Algorithm 2** **Prophecize**$(\langle X, I, T\rangle, P(X), t, n)$

**if** $n = 0$ **then**
  **return** $\langle\langle X \uplus \{p_t\}, I, T \cup \{p_t' = p_t\}\rangle, p_t = t \implies P(X), p_t\rangle$
**else**
  $\langle\langle X^h, I^h, T^h\rangle, h_t^n\rangle :=$ **Delay**$(\langle X, I, T\rangle, t, n)$
  **return** $\langle\langle X^h \uplus \{p_t^n\}, I, T \cup \{p_t^{n'} = p_t^n\}\rangle, p_t = h_t^n \implies P(X), p_t^n\rangle$
**end if**

**Theorem 3.** *Let $\mathcal{S} = \langle X, I, T\rangle$ be an STS, $P(X)$ a formula, and $t$ a term. Then, $\mathcal{S} \models P(X)$ iff $\mathcal{S} \models \forall\, y.(y = t \implies P(X))$, where $y$ is not free in $P(X)$.*

Theorems 2 and 3 are special cases of Theorems 3 and 4 of [52]. The original theorems handle the more general case where $P(X)$ can be a temporal formula.

## 3 Using Auxiliary Variables to Assist Induction

We can use Theorem 3 followed by Theorem 2 to introduce frozen prophecy variables that predict the value of a term $t$ when the property $P$ is being checked. We refer to $t$ as the prophecy *target* and the process as *universal* prophecy. If we also use **Delay**, we can target a term at some finite number of steps *before* the property is checked. This is captured by Algorithm 2, which takes a transition system, property $P(X)$, term $t$, and $n \geq 0$. If $n = 0$, it introduces a universal prophecy variable for $t$. Otherwise, it first introduces history variables for $t$ and then applies universal prophecy to the delayed $t$. In either case it returns the augmented system, augmented property, and the prophecy variable.

We will use the STS shown in Fig. 1(a) as a running example throughout the paper (it is inspired by the hardware example from [10]). We assume the background theory $\mathcal{T}$ includes integer arithmetic and arrays of integers indexed by integers. The variables in this STS include an array and four integer variables, representing the read index, write index, read data, and write data, respectively. The system starts with an array of all zeros. At every step, if the write data is less than 200, it writes that data to the array at the write index. Otherwise, the array stays the same. Additionally, the read data is updated with the current value of $a$ at $i_r$. This effectively introduces a one-step delay between when the value is read from $a$ and when the value is present in $d_r$. The property is that $d_r < 200$. This property is clearly true, but it is not straightforward to prove with standard model checking techniques because it is not inductive. Note that it is also not $k$-inductive for any $k$ [59]. The primary issue is that it does not constrain the value of $a$ at all, so in an inductive proof, the value of $a$ could be anything in the induction hypothesis.

One way to prove the property is to strengthen it with the quantified invariant: $\forall\, i.\ \mathit{read}(a, i) < 200$. Remarkably, observe that by augmenting the system using **Prophecize**, it is possible to prove the property using only a *quantifier-free* invariant. In this case, the relevant prophecy target is the value of $i_r$ one

$$I := a = \mathit{constarr}(\mathit{0}) \wedge d_r < 200$$
$$T := a' = \mathit{ite}(d_w < 200, \mathit{write}(a, i_w, d_w), a) \wedge d_r' = \mathit{read}(a, i_r)$$
$$P := d_r < 200$$

**(a)**

$$I := a = \mathit{constarr}(\mathit{0}) \wedge d_r < 200$$
$$T := a' = \mathit{ite}(d_w < 200, \mathit{write}(a, i_w, d_w), a) \wedge d_r' = \mathit{read}(a, i_r) \wedge {p^1_{i_r}}' = p^1_{i_r} \wedge {h^{\mathit{1}}_{i_r}}' = i_r$$
$$P := p^1_{i_r} = h^{\mathit{1}}_{i_r} \implies d_r < 200$$

**(b)**

**Fig. 1:** (a) Running example. (b) Running example with prophecy variable.

step before checking the property. We run **Prophecize**$(\langle X, I, T\rangle, P, i_r, 1)$ and it returns the system and property shown in Fig. 1(b), along with the prophecy variable $p^1_{i_r}$. This augmented system has a simple, quantifier-free invariant which can be used to strengthen the property, making it inductive: $\mathit{read}(a, p_{i_r}) < 200$. This formula holds in the initial state because of the constant array, and if we start in a state where it holds, it still holds after a transition.

Notice that the invariant learned over the prophecy variable has the same form as the original quantified invariant. However, we have instantiated that universal quantifier with a fresh, frozen prophecy variable. Intuitively, the prophecy variable captures a proof by contradiction: assume the property does not hold, consider the value of $i_r$ one step before the first failure of the property, and then use this value to show the property holds. This example shows that auxiliary variables can be used to transform an STS without a quantifier-free inductive invariant into an STS with one. However, it is not yet clear how to identify good targets for history and prophecy variables. In the next section, we show how this can be done as part of an abstraction refinement scheme for symbolic transition systems over the theory of arrays.

## 4 Abstraction Refinement for Arrays

We now introduce our main contribution. Given a background theory $\mathcal{T}_B$ and a model checking algorithm for STS's over $\mathcal{T}_B$, we use an instantiation of the CEGAR loop in Algorithm 1 to check properties of STS's over the theory that combines $\mathcal{T}_B$ and the theory of arrays, $\mathcal{T}_A$. The key idea is to abstract all array operators and then add array lemmas as needed during refinement.

**Abstract and Prove.** We use a standard abstraction for the theory of arrays, which we denote **Abstract-Arrays**. Every array sort is replaced with an uninterpreted sort, and the array variables are abstracted accordingly. Each constant array is replaced by a fresh abstract array variable, which is then constrained to be frozen (because constant arrays do not change over time). Additionally, we replace the *read* and *write* array operations with uninterpreted functions. Note that if the system contains multiple array sorts, we need to introduce a separate read and write function for each uninterpreted abstract array sort. Using uninterpreted sorts and functions for abstracting arrays is a common technique in Satisfiability Modulo Theories [7] (SMT) solvers [32]. Intuitively, our initial abstraction starts with *memoryless* arrays. We then incrementally refine the arrays'

$$\widehat{I} := \widehat{a} = \widehat{constarr0} \wedge d_r < 200$$
$$\widehat{T} := \widehat{a}' = ite(d_w < 200, \widehat{write}(\widehat{a}, i_w, d_w), \widehat{a}) \wedge$$
$$d_r' = \widehat{read}(\widehat{a}, i_r) \wedge \widehat{constarr0}' = \widehat{constarr0}$$
$$\widehat{P} := d_r < 200$$

**Fig. 2:** Result of calling **Abstract** on the example from Fig. 1(a)

memory as needed. Fig. 2 shows the result of running **Abstract-Arrays** on the example from Fig. 1(a). **Prove** can be instantiated with any (unbounded) model checker that can accept expressions over the background theory $\mathcal{T}_B$ combined with the theory of uninterpreted functions. In particular, due to our abstraction, the model checker does not need to support the theory of arrays.

**Refine.** Here, we explain the refinement approach for our array abstraction. At a high level, we solve a BMC problem over the abstract STS at bound $k$. We then look for violations of array axioms in the returned counterexample, and instantiate each violated axiom (this is essentially the same as the lazy array axiom instantiation approach used in SMT solvers [13,14,17,27]). We then *lift* these axioms to the STS-level by modifying the STS. It is this step that may require introducing auxiliary variables. The details are shown in Algorithm 3.

We start by computing a set $\mathcal{I}$ of index terms with *ComputeIndices* – this set is used in the lazy axiom instantiation step below. We add to $\mathcal{I}$ every term that appears in a $\widehat{read}$ or $\widehat{write}$ operation in $BMC(\widehat{\mathcal{S}}, \widehat{P}, k)$. We also add a witness index for every array equality - the witness corresponds to a skolemized existential variable in the contrapositive of axiom (ext). For soundness, we must add an extra variable $\lambda_\sigma$ for each index sort $\sigma$ and constrain it to be different from all the other index variables of the same sort (this is based on the approach in [13]). Intuitively, this variable represents an arbitrary index different from those mentioned in the STS. We assume that the index sorts are from an infinite domain so that a distinct element is guaranteed. For simplicity of presentation, we also assume from now on that there is only a single index sort (e.g. integers). Otherwise, $\mathcal{I}$ must be partitioned by sort. For the abstract STS in Fig. 2, with $k = 1$, the index set would be $\mathcal{I} := \{i_r@0, i_w@0, w_0@0, w_1@0, \lambda_{Int}@0, i_r@1, i_w@1, w_0@1, w_1@1, \lambda_{Int}@1\}$, where $w_0$ and $w_1$ are witness indices.

After computing indices, the algorithm enters the main loop. We first check the $BMC(\widehat{\mathcal{S}}, \widehat{P}, k)$ query. The result $\rho$ is either a counterexample, or the distinguished value $\bot$, indicating that the query is unsatisfiable. If it is the latter, then we return the refined STS and property, as the property now holds on the STS up to bound $k$. Otherwise, we continue. The next step (line 5) is to find violations of array axioms in the execution $\rho$ based on the index set $\mathcal{I}$.

*CheckArrayAxioms* takes two arguments, a counterexample and an index set, and returns instantiated array axioms that do not hold over the counterexample. This works as follows. We first look for occurrences of $\widehat{write}$ in the BMC formula.

**Algorithm 3 Refine-Arrays** $(\widehat{\mathcal{S}} \coloneqq \langle \widehat{X}, \widehat{I}, \widehat{T}\rangle, \widehat{P}, k)$

$\mathcal{I} \leftarrow \mathit{ComputeIndices}(\widehat{\mathcal{S}}, \widehat{P}, k)$
**loop**
  $\rho \leftarrow \mathrm{BMC}(\widehat{\mathcal{S}}, \widehat{P}, k)$
  **if** $\rho = \bot$ **then return** $\langle\langle \widehat{X}, \widehat{I}, \widehat{T}\rangle, \widehat{P}, \mathit{true}\rangle$ // Property holds up to bound $k$
  $\langle ca, nca\rangle \leftarrow \mathit{CheckArrayAxioms}(\rho, \mathcal{I})$
  **if** $ca = \emptyset \wedge nca = \emptyset$ **then return** $\langle\langle \widehat{X}, \widehat{I}, \widehat{T}\rangle, \widehat{P}, \mathit{false}\rangle$ // True counterexample
  // Go through non-consecutive array axiom instantiations
  **for** $\langle ax, i@n_i\rangle \in nca$ **do**
    **let** $n_{min} \coloneqq \mathit{min}(\tau(ax)\backslash\{n_i\})$
    $\langle\langle X^p, I^p, T^p\rangle, P^p, p_i^{k-n_i}\rangle \leftarrow$ **Prophecize**$(\langle \widehat{X}, \widehat{I}, \widehat{T}\rangle, \widehat{P}, i, k-n_i)$
    $ax_c \leftarrow ax\{i@n_i \mapsto p_i^{k-n_i}@n_{min}\}$
    $ca \leftarrow ca \uplus \{ax_c@n_{min}\}$ // add consecutive version of axiom
    $\mathcal{I} \leftarrow \mathcal{I} \uplus \{p_i^{k-n_i}@0, \ldots, p_i^{k-n_i}@k\}$
    $\widehat{X} \leftarrow X^p$; $\widehat{I} \leftarrow I^p$; $\widehat{T} \leftarrow T^p$; $\widehat{P} \leftarrow P^p$
  **end for**
  // Go through consecutive array axiom instantiations
  **for** $ax \in ca$ **do**
    **let** $n_{min} \coloneqq \mathit{min}(\tau(ax))$, $n_{max} \coloneqq \mathit{max}(\tau(ax))$
    assert$(n_{max} = n_{min} \vee n_{max} = n_{min} + 1)$
    **if** $k = 0$ **then**
      $\widehat{I} \leftarrow \widehat{I} \wedge ax\{X@n_{min} \mapsto X\}$
    **else if** $n_{min} = n_{max}$ **then**
      $\widehat{T} \leftarrow \widehat{T} \wedge ax\{X@n_{min} \mapsto X\} \wedge ax\{X@n_{min} \mapsto X'\}$
    **else**
      $\widehat{T} \leftarrow \widehat{T} \wedge ax\{X@n_{min} \mapsto X\}\{X@(n_{min}+1) \mapsto X'\}$
    **end if**
  **end for**
**end loop**

For each such occurrence, we instantiate the (write) axiom so that the $\widehat{\mathit{write}}$ term in the axiom matches the term in the formula (i.e., we use the $\widehat{\mathit{write}}$ term as a trigger). This instantiates all quantified variables except for $i$. We then instantiate $i$ once for each variable in the index set. We evaluate each of the instantiated axioms using the values from the counterexample and keep those instantiations that reduce to false. We do the same thing for the (const) axiom, using each constant array term in the BMC formula as a trigger. Finally, for each array equality $a@m = b@n$ in the BMC formula, we check an instantiation of the contrapositive of (ext): $a@m \neq b@n \rightarrow \mathit{read}(a@m, w_i@n) \neq \mathit{read}(b@n, w_i@n)$. We add instantiated formulas that do not hold in $\rho$ to the set of violated axioms.

*CheckArrayAxioms* sorts the collected axiom instantiations into two sets based on which timed variables they contain. The *consecutive* set contains formulas with timed variables whose timing differs by at most one; whereas the timed variables in the formulas contained in the *non-consecutive* set may differ by more. Formally, let $\tau$ be a function which takes a single timed variable and returns its time (e.g., $\tau(i@2) = 2$). We lift this to formulas by having $\tau(\phi)$ return the set of all time-steps for variables in $\phi$. A formula $\phi$ is *consecutive* iff $max(\tau(\phi)) - min(\tau(\phi)) \leq 1$. Note that instantiations of (ext) are consecutive by construction. Additionally, because constant arrays have the same value in all time steps, we can always choose a representative time step for instantiations of (const) that results in a consecutive formula. However, instantiations of (write) may be non-consecutive, because the variable from the index set may be from a time step that is different from that of the trigger term. *CheckArrayAxioms* returns the pair $\langle ca, nca \rangle$, where $ca$ is a set of consecutive axiom instantiations and $nca$ is a set of pairs – each of which contains a non-consecutive axiom instantiation and the index-set variable that was used to create that instantiation.

At line 6, we check if the returned sets are empty. If so, then there are no array axiom violations and $\rho$ is a concrete counterexample. In this case, the system, property, and *false* are returned. Otherwise, we process the two sets. In lines 8-15, we process the non-consecutive formulas. Given a non-consecutive formula $ax$ together with its index-set variable $i@n_i$, we first compute the minimum time-step of the axiom's other variables, $n_{min}$. We then use the **Prophecize** method to create a prophecy variable $p_i^{k-n_i}$, that is effectively a way to refer to $i@n_i$ at time-step $n_{min}$ (line 10). This allows us to create a consecutive formula $ax_c$ that is semantically equivalent to $ax$ (line 11). This new consecutive formula is added to $ca$ in line 12, and in line 13 the introduced prophecy variables (one for each time-step) are added to the index set. Then, line 14 updates the abstraction.

At line 17, we are left with a set of consecutive formulas to process. For each consecutive formula $ax$, we compute the minimum and maximum time-step of its variables (line 18), which must differ by no more than 1 (line 19). There are three cases to consider: i) when $k = 0$, the counterexample consists of only the initial state–we thus refine the initial state by adding the untimed version of $ax$ to $\widehat{I}$ (line 21); ii) if $ax$ contains only variables from a single time step, then we add the untimed version of $ax$ as a constraint for both $X$ and $X'$, ensuring that it will hold in every state (line 23); iii) finally, if $ax$ contains variables from two adjacent time steps, we can translate this directly into a transition formula to be added to $\widehat{T}$ (line 25). The loop then repeats with the newly refined STS.

*Example.* Consider again the example from Fig. 2, and suppose **Refine-Arrays** is called on $\widehat{\mathcal{S}}$ and $\widehat{P}$ with $k = 3$. At this unrolling, one possible abstract counterexample violates the following nonconsecutive axiom instantiation:

$$(i_r@2 = i_w@0 \implies \widehat{read}(\widehat{write}(\widehat{a@0}, i_w@0, d_w@0), i_r@2) = d_w@0) \land$$
$$(i_r@2 \neq i_w@0 \implies \widehat{read}(\widehat{write}(\widehat{a@0}, i_w@0, d_w@0), i_r@2) = \widehat{read}(\widehat{a}@0, i_r@2))$$

Calling **Prophecize**$(\widehat{\mathcal{S}}, \widehat{P}, i_r, 1)$ returns the new STS $\langle\langle \widehat{X} \uplus \{h_{i_r}^{1}, p_{i_r}^1\}, \widehat{I}, \widehat{T} \land h_{i_r}^{1\,\prime} = i_r \land p_{i_r}^{1\,\prime} = p_{i_r}^1 \rangle$ and the new property $p_{i_r}^1 = h_{i_r}^{1} \implies d_r < 200$. The history variable $h_{i_r}^{1}$ makes the previous value of $i_r$ available at each time-step, and the prophecy variable $p_{i_r}^1$ mimics a universally quantified variable. We substitute $p_{i_r}^1@0$ for $i_r@2$ to obtain a consecutive formula. Its untimed version (and a primed version) is added to the transition relation.

We stress that processing nonconsecutive axioms using **Prophecize** is how we automatically discover the universal prophecy variable $p^1_{i_r}$, and it is exactly the universal prophecy variable that was needed in Sec. 3 to prove correctness of the running example. An alternative approach could avoid nonconsecutive axioms using Craig interpolants [26] so that only consecutive axioms are found [15]. However, quantifier-free interpolants are not guaranteed to exist for the standard theory of arrays, and the auxiliary variables found using nonconsecutive axioms are needed to improve the chances of finding a quantifier-free inductive invariant.

It is important to have enough prophecy variables to assist in constructing inductive invariants. We found that we could often obtain a larger, richer set of prophecy variables by weakening our array abstraction. We do this by replacing equality between arrays by an uninterpreted predicate, and also checking the congruence axiom, the converse of (ext). Since more axioms are checked, there are more opportunities to introduce auxiliary variables. We call this *weak* abstraction (**WA**) as opposed to *strong* abstraction (**SA**), which uses regular equality between abstract arrays and guarantees congruence through UF axioms.

On the other hand, an excessive number of unnecessary auxiliary variables could overwhelm the **Prove** step. Thus, an improvement not shown in Algorithm 3 is to check consecutive axioms first and only add nonconsecutive ones when necessary. This is the motivation behind the custom array solver implementation *CheckArrayAxioms* based on [13]. In principle, we could have used an SMT solver to find array axioms, but it would give no preference to consecutive axioms. Similarly, we could overwhelm the algorithm with unnecessary consecutive axioms. *CheckArrayAxioms* can still produce hundreds or even thousands of (consecutive) axiom instantiations. Once these are lifted to the transition system, some may be redundant. To mitigate this issue, when the BMC check returns $\perp$ and we are about to return (line 4), we keep only axioms that appear in the unsat core of the BMC formula [22].

**Correctness.** We now state two important correctness theorems. Note that here and below, proofs are omitted due to space constraints. An extended version with proofs is available at: https://arxiv.org/abs/2101.06825.

**Theorem 4.** *Algorithm 1, instantiated with* ***Abstract-Arrays****, a model-checker* ***Prove*** *as described above, and* ***Refine-Arrays*** *is sound.*

**Theorem 5.** *If Algorithm 1, instantiated with* ***Abstract-Arrays****,* ***Prove*** *as described above, and* ***Refine-Arrays****, returns false, there is a concrete counterexample of length $k$ in the concrete transition system.*

## 5 Expressiveness and Limitations

We now address the expressiveness of counterexample-guided prophecy with regard to the introduction of auxiliary variables. For simplicity, we ignore the array abstraction, relying on the correctness theorems. An inductive invariant using auxiliary variables can be converted to one without auxiliary variables by first universally quantifying over the prophecy variables, then existentially quantifying over the history variables. The details are captured by this theorem:

**Theorem 6.** *Let* $\mathcal{S} := \langle X, I, T\rangle$ *be an STS, and* $P(X)$ *be a property such that* $\mathcal{S} \models P(X)$. *Let* $H$ *be the set of history variables, and* $\mathcal{P}$ *be the set of prophecy variables introduced by* ***Refine-Arrays***. *Let* $\tilde{\mathcal{S}} := \langle X \cup H \cup \mathcal{P}, I, \tilde{T}\rangle$ *and* $\tilde{P} := (\bigwedge_{p\in\mathcal{P}} p = \tilde{t}(p)) \implies P(X)$ *be the system and property with auxiliary variables. The function* $\tilde{t}$ *maps prophecy variables to their target term from* ***Prophecize***. *If* $Inv(X, H, \mathcal{P})$ *is an inductive invariant for* $\tilde{\mathcal{S}}$ *and entails* $\tilde{P}$, *then* $\exists H \forall \mathcal{P} Inv(X, H, \mathcal{P})$ *is an inductive invariant for* $\mathcal{S}$ *and entails* $P$, *where* $\exists H$ *and* $\forall \mathcal{P}$ *bind each variable in the set with the corresponding quantifier.*

Although the invariants found using counterexample-guided prophecy correspond to $\exists\forall$ invariants over the unmodified system, we must acknowledge that the existential power is very weak. The existential quantifier is only used to remove history variables. While history variables can certainly be employed for existential power in an invariant [55], these specific history variables are introduced solely to target a term for prophecy and only save a term for some fixed, finite number of steps. Thus, we do not expect to gain much existential power in finding invariants on practical problems. This use of history and prophecy variables can be thought of as quantifier instantiation at the model checking level, where the instantiation semantically uses a term appearing in an execution of the system. Consequently, our technique performs well on systems where there is only a small number of instantiations needed over terms that are not too distant in time from a potential property violation that must be disproved (i.e., not many history variables are required). This appears to be a common situation for invariant-finding benchmarks, as we show empirically in Sec. 6.

**Limitations.** If our CEGAR loop terminates, it either terminates with a proof or with a true counterexample. However, it is possible that the procedure may not terminate. In particular, while we can always refine the abstraction for a given bound $k$, there is no guarantee that this will eventually result in a refinement that rules out all spurious counterexamples (of any length).

This failure mode occurs, for instance, when no finite number of instantiations can capture all the relevant indices of the array. Consider an example system with $I := a = \mathit{constarr}(0)$, $T := a' = \mathit{write}(a, i_0, \mathit{read}(a, i_1) + 1)$, and $P := \mathit{read}(a, i_r) \geq 0$. The array $a$ is initialized with 0 at every index, and at every step, $a$ is updated at a single index by reading from an arbitrary index of $a$ and adding 1 to the result. Note that the index variables are unconstrained: they can range over the integers freely at each time step. Then, the property is that every element of $a$ is positive. This property clearly holds because of a quantified invariant maintained by the system: $\forall i \;.\; \mathit{read}(a, i) \geq 0$.

However, the initial abstraction is a memoryless array which can easily violate the property by returning negative values from reads. Since the array is updated in each step at an arbitrary index based on a read from another arbitrary index, no finite number of prophecy variables can capture all the relevant indices. It will successively rule out longer finite spurious counterexamples, but will never be refined enough to prove the property unboundedly. We believe that this limitation can be addressed in future work, perhaps by adapting techniques from [52]. However, it is not yet clear how to automate that process. Note that an even simpler system which does not add 1 in the update would already be problematic; however, for that case, it is straightforward to extend our algorithm to have it learn that the array does not change.

A related, but less fundamental issue is that the index set might not contain the best choice of targets for prophecy. While the index set *is* sufficient for ruling out bounded counterexamples, it is possible there is a better target for universal prophecy that does not appear in the index set. However, based on the evaluation in Sec. 6, it appears that the index set does work well in practice.

## 6 Experiments

**Implementation.** In this section, we evaluate a prototype implementation of counterexample-guided prophecy, which instantiates **Prove** with `ic3ia` [34] (downloaded Apr 27, 2020), an open-source C++ implementation of IC3 via Implicit Predicate Abstraction (IC3IA) [20], which is itself a CEGAR loop that uses implicit predicate abstraction to perform IC3 [12] on infinite-state systems and uses interpolants to find new predicates. `ic3ia` uses MathSAT [21] (version 5.6.3) as the backend SMT solver and interpolant producer. We call our prototype `prophic3` [48]. In our implementation, we also include a simple abstraction-refinement wrapper which abstracts large constant integers and refines them with the actual values if that fails. This is especially useful for dealing with software benchmarks with large constant loop bounds. Otherwise, the system might need to be unrolled to a very large bound to reach an abstract counterexample.

**Setup.** We evaluate our tool against three state-of-the-art tools for inferring universally quantified invariants over linear arithmetic and arrays: `freqhorn`, `quic3`, and `gspacer`. All these tools are Constrained Horn Clause (CHC) solvers built on Z3 [54]. The algorithm implemented in `freqhorn` [28] is a *syntax-guided synthesis* [4] approach for inferring universally quantified invariants over arrays [29]. `quic3` is built on Spacer [40], the default CHC engine in Z3, and extends IC3 over linear arithmetic and arrays to allow universally quantified frames (frames are candidates for inductive invariants maintained by the IC3 algorithm). It also maintains a set of quantifier instantiations which are provided to the underlying SMT solver. `quic3` was recently incorporated into Z3. We used Z3 version 4.8.9 with parameters suggested by the `quic3` authors.[4] Finally, `gspacer` is an extension of Spacer which adds three new inference rules for improving local generalizations with global guidance. While this last technique does not specifically target universally quantified invariants, it can be used along with the `quic3` options in Spacer and potentially executes a much different search. The `gspacer`

[4] `fp.spacer.q3.use_qgen=true fp.spacer.ground_pobs=false fp.spacer.mbqi=false fp.spacer.use_euf_gen=true`

| group | freqhorn (81) | quic3 (42) | vizel (32) | | chc-comp (501) | | tool total | |
|---|---|---|---|---|---|---|---|---|
| `prophic3` | **67**/4 | **42**/0 | **20**/3 | 1 | 43/159 | 59 | 172/166 | 60 |
| `prophic3-SA` | 62/4 | 37/0 | 19/3 | 1 | 36/**160** | 67 | 154/**167** | 68 |
| `freqhorn` | 65/4 | 0/0 | 0/1 | 0 | 5/46 | 1 | 70/51 | 1 |
| `quic3` | 55/4 | 34/0 | 15/4 | 1 | **74**/137 | 75 | **178**/145 | 76 |
| `gspacer` | 35/**5** | 27/0 | 18/4 | 1 | 66/138 | **94** | 146/147 | **95** |
| `ic3ia` | 0/4 | 0/0 | 0/3 | 1 | 0/158 | 59 | 0/165 | 60 |
| spacer | 0/5 | 0/0 | 0/4 | 1 | 0/134 | 77 | 0/143 | 78 |

**Fig. 3:** Experimental results. The safe results are reported as # *Q* / # *QF*. The second column per group shows unsafe results, the first two groups had only safe benchmarks.

submission [43] won the arrays category in CHC-COMP 2020 [58]. We also include `ic3ia` and the default configuration of Spacer in our results, neither of which can produce universally quantified invariants. Our default configuration of `prophic3` uses weak abstraction, but we also include a version running strong abstraction (`prophic3-SA`) in our experiments. We chose to build our prototype on `ic3ia` instead of Spacer, in part because we needed uninterpreted functions for our array abstraction, and Spacer does not handle them in a straightforward way, due to the semantics of CHC [11].

We compare these solvers on four benchmark sets: i) *freqhorn* - benchmarks from the `freqhorn` paper [29]; ii) *quic3* - benchmarks from the `quic3` paper [37] (these were C programs from SV-COMP [8] that were modified to require universally quantified invariants); iii) *vizel* - additional benchmarks provided to us by the authors of [37]; and iv) *chc-comp-2020* - the array category benchmarks of CHC-COMP 2020 [57]. Additionally, we sort the benchmarks into three categories: 1) Q - safe benchmarks solved by some tool supporting quantified invariants but none of the solvers that do not; 2) QF - those solved by at least one of the tools that do not support quantified invariants, plus any unsafe benchmarks; and 3) U - unsolved benchmarks. Because not all of the benchmark sets were guaranteed to require quantifiers, this is an approximation of which benchmarks required quantified reasoning to prove safe.

Both `prophic3` and `ic3ia` take a transition system and property specified in the Verification Modulo Theories (VMT) format [23], which is a transition system format built on SMT-LIB [6]. All other solvers read the CHC format. We translated benchmark sets i and iv from CHC to VMT using the *horn2vmt* program which is distributed with `ic3ia`. For benchmark sets ii and iii, we started with the C programs and generated both VMT and CHC using *Kratos2* (an updated version of *Kratos* [19]). We ran all experiments on a 3.5GHz Intel Xeon E5-2637 v4 CPU with a timeout of 2 hours and a memory limit of 32Gb. An artifact for reproducing these results is publicly available [49,38].

**Results.** The results are shown in Fig. 3. We first observe that `prophic3` solves the most benchmarks in each of the first three sets, both overall and in category Q. The quic3 (and most of the freqhorn) benchmarks require quantified invariants; thus, `ic3ia` and Spacer cannot solve any of them. On solved instances in the Q category, `prophic3` introduced an average of 1.2 prophecy variables and a median of 1. This makes sense because, upon inspection, most benchmarks only require one quantifier and we are careful to only introduce prophecy variables when needed. On benchmarks it cannot solve, `ic3ia` either times out or fails to compute an interpolant. This is expected because quantifier-free interpolants are not guaranteed over the standard theory of arrays. Even without arrays, it is also possible for `prophic3` to fail to compute an interpolant, because MathSAT's interpolation procedure is incomplete for combinations with non-convex theories such as integers. However, this was rarely observed in practice.

We also observe that `prophic3-SA` solves fewer benchmarks in the first three sets. However, it is faster on commonly solved instances. This makes sense because it needs to check fewer axioms (it uses built-in equality and thus does not check equality axioms). We suspect that it solves fewer benchmarks in the first three sets because it was unable to find the right prophecy variable. For example, for the `standard_find_true-unreach-call_ground` benchmark in the *quic3* set, a prophecy variable is needed to find a quantifier-free invariant. However, because of the stronger reasoning power of **SA**, the system can be sufficiently refined without introducing auxiliary variables. `ic3ia` is then unable to prove the property on the resulting system without the prophecy variable, instead timing out. Interestingly, notice that `prophic3-SA` solves the most benchmarks in the QF category overall, suggesting that there are practical performance benefits of the CEGAR approach even when quantified reasoning is not needed.

There was one discrepancy on the CHC-COMP 2020 benchmarks: `gspacer` disagrees with `quic3`, Spacer, and `prophic3` on *chc-LIA-lin-arrays_381*. This is the same discrepancy mentioned in the CHC-COMP 2020 report [58]. `prophic3` proved this benchmark safe without introducing any auxiliary variables and we used both CVC4 [5] and MathSAT to verify that the solution was indeed an inductive invariant for the concrete system. We are confident that this benchmark is safe and thus do not count it as a solved instance for `gspacer`.

Some of the tools are sensitive to the encoding. Since it is syntax-guided, `freqhorn` is sensitive to the encoding syntax. The freqhorn benchmarks were hand-written to be syntactically simple, an encoding which is also good for `prophic3`. However, `prophic3` can be sensitive to other encodings. For example, the quic3 benchmarks are also included in the *chc-comp-2020* set, but translated by `SeaHorn` [35] instead of *Kratos2*. `prophic3` does much worse on the `SeaHorn` encoding (6 vs 42). We stress that the CHC solvers performed similarly on both encodings, so we did not compare against disadvantaged solvers. In fact, `quic3` and `freqhorn` solved exactly the same number in both translations. However, `gspacer` solved fewer using the *Kratos2* encoding (27 vs 34). Importantly, `prophic3` on the *Kratos2* encoding solved more benchmarks than any other tool and encoding pair.

There are two main reasons why `prophic3` fails on the `SeaHorn` encodings. First, due to the LLVM-based encoding, some of the `SeaHorn` translations have index sets which are insufficient for finding the right prophecy variable. This has to do with the memory encoding and the way that fresh variables and guards are used. `SeaHorn` also splits memories into ranges which is problematic for our technique. Second, the SeaHorn translation is optimized for CHC, not for transition systems. For example, it introduces many new variables, and the argument order between different predicates may not match. In the transition system, this essentially has the effect of interchanging the values of variables between each loop. SeaHorn has options that address some of these issues, and these helped prophic3 solve more benchmarks, but none of these options produce encodings that work as well as the *Kratos2* encodings. The difference between good CHC and transition system encodings could also explain the overall difference in performance on *chc-comp-2020* benchmarks, most of which were translated by SeaHorn. Both of these issues are practical, not fundamental, and we believe they can be resolved with additional engineering effort.

## 7 Related Work

There are two important related approaches for abstracting arrays in horn clauses [53] and memories in hardware [10]. Both make a similar observation that arrays can be abstracted by modifying the property to maintain values at only a finite set of symbolic indices. We differ from the former by using a refinement loop that automatically adjusts the precision and targets relevant indices. The latter is also a refinement loop that adjusts precision, but differs in the domain and the refinement approach, which uses a multiplexer tree. We differ from both approaches in our use of array axioms to find and add auxiliary variables.

A similar lazy array axiom instantiation technique is proposed in [15]. However, their technique utilizes interpolants for finding violated axioms and cannot infer universally quantified invariants. The work of [18] also uses lazy axiom-based refinement, abstracting non-linear arithmetic with uninterpreted functions. We differ in the domain and the use of auxiliary variables. In [55], prophecy variables defined by temporal logic formulas are used for liveness and temporal proofs, with the primary goal of increasing the power of a temporal proof system. In contrast, we use prophecy variables here for a different purpose, and we also find them automatically. The work of [24] includes an approach for synthesizing auxiliary variables for modular verification of concurrent programs. Our approach differs significantly in the domain and details.

There is a substantial body of work on automated quantified invariant generation for arrays using first-order theorem provers [42,16,41,51]. These include extensions to saturation-based theorem proving to analyze specific kinds of predicates, and an extension to paramodulation-based theorem proving to produce universally quantified interpolants. In [46], the authors propose an abstract interpretation approach to synthesize universally quantified array invariants. Our method also uses abstraction, but in a CEGAR framework.

Two other notable approaches capable of proving properties over arrays that require invariants with alternating quantifiers are [30,56]. The former proposes *trace logic* for extending first-order theorem provers to software verification, and the latter takes a *counterexample-guided inductive synthesis* approach. Our approach takes a model checking perspective and differs significantly in the details.

While these approaches are more general, we compared against state-of-the-art tools that focus specifically on universally quantified invariants.

MCMT [31,33,25] and its derivatives [2,3] are backward-reachability algorithms for proving properties over "array-based systems," which are typically used to model parameterized protocols. These approaches target syntactically restricted *functional* transition systems with universally quantified properties, whereas our approach targets general transition systems. Two other approaches for solving parameterized systems modeled with arrays are [36] and [47]. The former iteratively fixes the number of expected universal quantifiers, then eagerly instantiates them and encodes the invariant search to nonlinear CHC. The latter first uses a finite-state model checker to discover an inductive invariant for a specific parameterization and then applies a heuristic generalization process. We differ from all these techniques in domain and the use of auxiliary variables. Due to the limitations explained in Sec. 5, we do not expect our approach to work well for parameterized protocol verification without improvements.

In [45], heuristics are proposed for finding predicates with free indices that can be universally quantified in a predicate abstraction-based inductive invariant search. Our approach is counterexample-guided and does not utilize predicate abstraction directly (although IC3IA does). The authors of [39] propose a technique for Java programs that associates heap memory with the program location where it was allocated and generates CHC verification conditions. This enables the discovery of invariants over all heap memory allocated at that location, which implicitly provides quantified invariants. This is similar to our approach in that it gives quantification power without explicitly using quantifiers and in that their encoding removes arrays. However, we differ in that we focus on transition systems and utilize a different paradigm to obtain this implicit quantification.

## 8 Conclusion

We presented a novel approach for model checking transition systems containing arrays. We observed that history and prophecy variables can be extremely useful for reducing quantified invariants to quantifier-free invariants. We demonstrated that an initially weak abstraction in our CEGAR loop can help us to *automatically* introduce relevant auxiliary variables. Finally, we evaluated our approach on four sets of interesting array-manipulating benchmarks. In future work, we hope to improve performance, explore a tighter integration with the underlying model checker, address the limitations described in Sec. 5, and investigate applications of counterexample-guided prophecy to other theories.

**Acknowledgments.** This work was supported by the National Science Foundation Graduate Research Fellowship Program under Grant No. DGE-1656518. Any opinions, findings, and conclusions or recommendations expressed in this material are those of the author(s) and do not necessarily reflect the views of the National Science Foundation. Additional support was provided by DARPA, under grant No. FA8650-18-2-7854. We thank these sponsors for their support. We would also like to thank Alessandro Cimatti for his invaluable feedback on the initial ideas of this paper.
